# Physical dosimetry reconstructions of significant radiation exposure at an industrial accelerator facility in Tianjin (China)

**DOI:** 10.1093/jrr/rrz072

**Published:** 2019-12-10

**Authors:** Shuzhou Ruan, Menghui Huo, Kaijun Su, Yulian Liu, Changxin Yan, Wenyi Zhang, Ling Jiao

**Affiliations:** Institute of Radiation Medicine, Chinese Academy of Medical Science & Peking Union Medical College, No.238 baidi road, Nankai District, Tianjin, China

**Keywords:** Accelerators, radiation accident, physical dose, dose estimation

## Abstract

The goal of this thesis is to estimate the physical radiation doses for two victims who were accidently exposed to an industrial electron beam at an industrial accelerator facility on 7 July 7 2016 in Tianjin, China. On the basis of the radiation source parameters, irradiation situation and irradiation time, physical dose reconstruction was carried out at the accident site by using a Bottle-Manikin-Absorption (BOMAB) phantom and an Alderson Radiation Therapy (ART) phantom. With thermoluminscent dosimeters (TLDs), skin estimation was conducted for the feet, calves, upper arms, left side of the body and neck, and the mean dose was estimated to be 14.1 ± 5.6 Gy. The foot and leg skin received the highest dose, which was >16.3 Gy. In addition, the mean dose estimated for the eye lens was 0.18 ± 0.07 Gy. The organ effective dose estimated and the total organs effective dose estimated were 0.46–4.94 mSv and 0.21 Sv, respectively. In the course of the accident, the damage caused by the electron radiation field to the exposed person was mainly to the skin, and the contributions to other radiation-sensitive organs were small. The damage to the organs other than the skin was mainly caused by the X-rays generated by the bremsstrahlung of the electron beam from the environment or the human body.

## INTRODUCTION

With the development of nuclear science, the application of this field is becoming increasingly extensive, which benefits society. However, there have been many radiation accidents around the world. In December 1991, a radiological accident occurred at an industrial accelerator in MD, USA [1]. From 3 June to 3 August 2006, radiological accidents occurred in Dakar and Abidjan, France [2]. In April 2009, a worker accidentally picked up a radioactive source in South America and was irradiated [3]. In Thailand, ten victims were exposed to varying levels of ionizing radiation, three of them died from severe exposure [4]. In addition, on 3 May 2014, a radioactive source was lost in Nanjing, China and a sanitation worker was severely irradiated due to accidentally picking up the source [5]. After a radiological accident, the irradiation doses should be estimated as soon as possible to provide an important basis for timely medical treatment and prognosis. Rapid estimation of the physical doses to interrelated organs and the skin is very important for accident characterization and clinical treatment.

There are several methods for estimating post-accident dose. Biological dose estimation is a relatively reliable method for whole-body irradiation [6], but the error in these dose estimation results can be high for partial irradiation of certain patients. Currently, the only feasible method for early and rapid dose estimation for patients exposed to local accidental high-dose radiation is physical dose calculation and measurement with detailed dosimetry parameters. Previous experience shows that physical dose estimation can reflect the effects of the damage and the course of disease, providing early and rapid judgement of the severity of injury inflicted on a patient irradiated in an accident and the whole body dose.

On 7 July 2016, an electron beam accelerator radiological accident occurred in the Binhai New Area of Tianjin, China. At approximately 17:00 hours, when the accelerator operators left the operating room for dinner, two temporary repair workers entered the irradiation chamber to repair a damaged cooling water pipe and checked the cooling water pipe at position A without informing the operators. At 17:35 hours, the accelerator operator turned the accelerator on without checking the irradiation chamber after finishing dinner. The maintenance workers noticed that the accelerator had activated and quickly walked out of the irradiation chamber. However, before the workers exited, the accelerator had begun to operate, and the workers were exposed to accidental ionizing radiation. The workers soon developed pain, numbness, burning and other symptoms in the left side of their limbs after the accidental exposure to radiation. The company immediately sent two workers to Beijing 307 Hospital for treatment. Due to the complex environmental conditions at the scene of the accident, it is very difficult to perform dose reconstruction using theoretical calculations and Monte Carlo simulation methods. Additionally, the biological dose estimation method of chromosome aberration in peripheral blood lymphocytes yielded normal results. Thus, we chose to reconstruct the dose with a simulation module in a field experiment.

## MATERIALS AND METHODS

### Description of the accident

**Fig. 1:**
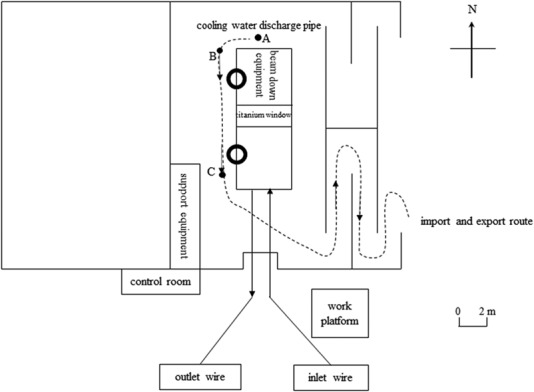
Plan of the accident accelerator chamber.

The layout of the acceleration chamber and the route of workers leaving the machine room are shown in Fig. 1. The monitoring video in the machine room shows that the staff remained at point A for 50 s after the accelerator was activated, and the workers walked from position B to position C between 50 and 60 s. The distance from point B to point C is 10 m. Finally, the two maintenance workers rapidly walked out of the machine room. The two men were not wearing personal dosimeters or any other protective equipment when they entered the irradiation chamber.

The irradiation system consists of two wheels on both sides, and the distance between the wheel edges is 3.8 m. A titanium window is located in the middle. The length of the titanium window is 1.5 m (including a 0.8 m beam spot), and the width is 0.2 m. Between the accelerator and the conveyor belt are the irradiated products, which during the accident were 6-m^2^ cables. The shortest distance between the walking route and the center of the titanium window is 2.2 m. The straight-line distance of a human walking is 10.0 m.

**Fig. 2:**
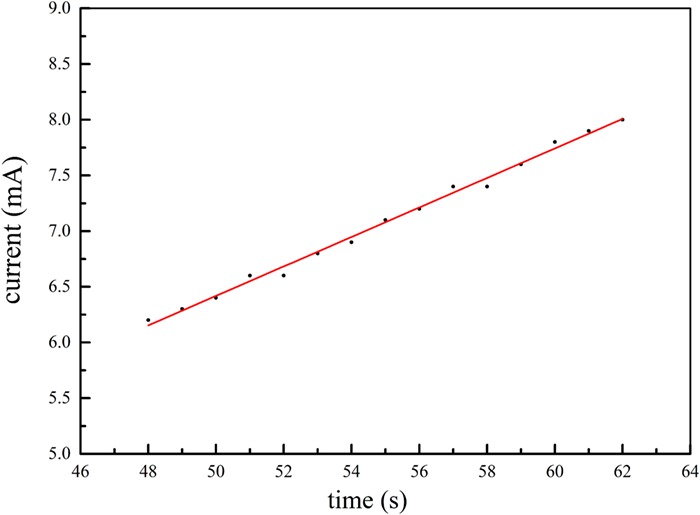
Start-up time(s) vs current (mA) for the accelerator within 48–62 s after power-on.

The radiation emitted by the accelerator in the event of a radiation accident is electron radiation. The trend of the current of the accelerator with startup time was obtained. The current of the accelerator was increasing during the irradiation of the workers and had not reached the rating. Figure 2 shows that between 50 and 60 s the current changes linearly with time under a voltage.

### Materials

The accelerator at the scene of the accident was an electron accelerator (AB 2.5-40-1200, Wuxi Aibang Radiation Technology Co., Ltd.). The electron energy of the accelerator is 2.2 MeV, and the full power operating current is 16 mA. The main source of radiation in the accident was X-rays from electrons and bremsstrahlung.

A thermoluminescent dosimeter detector annealing furnace (BR2000A, Beijing Bo-Chuangte technology development Co., Ltd.), thermoluminescent personal dosimeters (MODEL469), GR-200aLiF (Mg, Cu, P) thermoluminescent detectors (TLDs), 1 mm polyethylene plastic, 5 mm acrylonitrile butadiene styrene (ABS) plastic, and a thermoluminescent dosimeter (FJ427A1, China National Nuclear Corporation (Beijing) nuclear instrument factory) were used.

An Alderson Radiation Therapy (ART) phantom represents an adult and has holes for TLDs. The ART phantom used herein comprised 35 numbered sections that represented the trunk of a man 175 cm tall with a mass of 73.5 kg. [7]. A Bottle-Manikin-Absorption (BOMAB) phantom represents an adult male human body. An ART phantom only has the main part of the body including the internal organs, without limbs. Thus, in the simulation process, we used a combination of an ART phantom and a BOMAB phantom.

The electronic calibration coefficients of the thermoluminescent dosimeters were given by the Chinese Academy of Radiation Protection as 1.61 × 10^−6^ Gy/count, and the relative extended uncertainty was 13%. The radiation calibration sources were ^90^Sr and ^90^Y, and the maximum electron energy was 2.274 MeV. The photon calibration coefficient was 1.37 × 10^−7^ Gy/count, and the relative extended uncertainty was 9%.

## Methods

### Estimating the radiation distribution on site

**Fig. 3:**
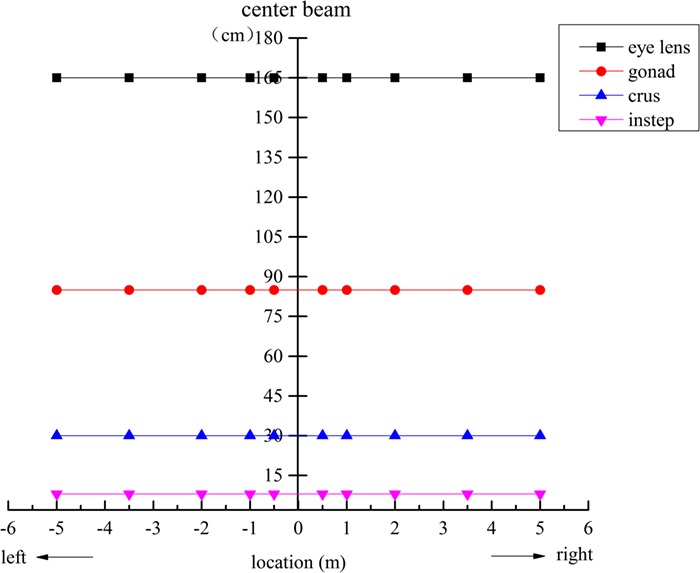
Thermoluminescent personal dosimeter suspension distance from the center beam (distance (m) and height (cm)) at the simulated accident site.

We took the center beam as the abscissa origin, with the left side as negative and the right side as positive. Thermoluminescent personal dosimeters were hung at different heights (8, 30, 85 and 165 cm) and positions (50, 100, 200, 350 and 500 cm) to estimate the beam field distribution at 50 and 60 s after the accelerator was turned on. As shown in Fig. 3, these heights represented different body parts including the instep (8 cm), crus (30 cm), gonad (85 cm) and eye lens (165 cm). We measured a total of 10 points (50, 100, 200, 350 and 500 cm) on the left and right sides.

### Experimental physical dosimetric reconstruction

During the simulation experiment, five TLDs were placed in a predetermined hole for the tissues and organs of the ART phantom. These organs and tissues were the liver, thyroid gland, lung, heart and abdomen. The TLDs were covered with 3 mm polyethylene plastic at the position of the eyes. Other TLDs were covered with 1 mm polyethylene plastic and 5 mm ABS plastic at the skin positions of the BOMAB phantom. The locations of the dosimeters included the upper left arm, left forearm, left thigh, left calf and feet. After the dosimeters were pasted on the phantom as required, the left side was placed close to the ray beam, approximately 2.0 m away from the center of the ray beam.

The accelerator was turned on, allowed to operate for 50 s, and shut off, and the values of the dosimeters at each position were read and recorded. The dosimeters were replaced with new ones at the corresponding positions. The accelerator was turned on, allowed to operate for 60 s, and shut off, and the procedure for the dosimeters was the same as that described above.

## RESULTS

### Doses distribution at the accident site

**Fig. 4:**
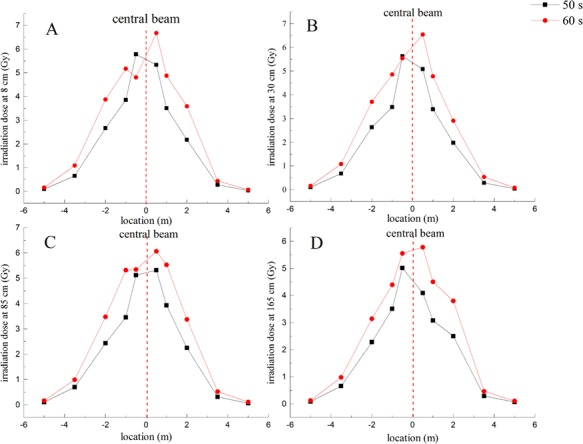
Dose distribution from the center beam distance at 50 and 60 s. (**A**, **B**, **C** and **D** show dose values for ten different locations at 8, 30, 85 and 165 cm, respectively.

**Fig. 5:**
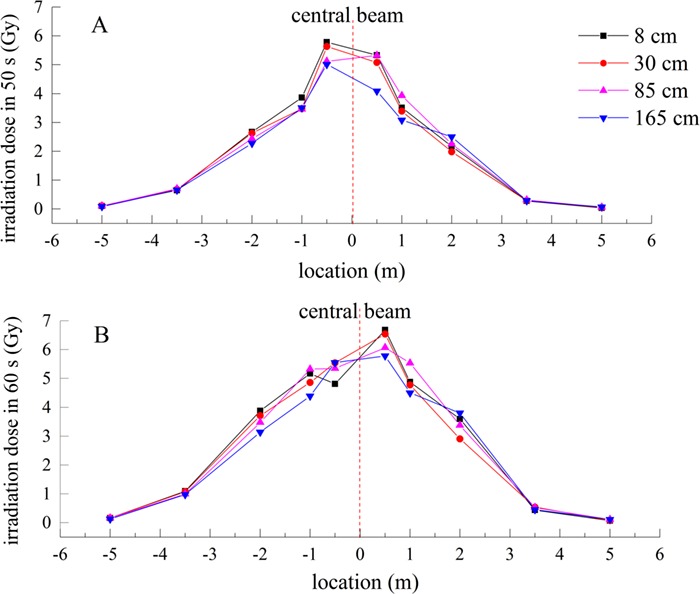
Comparison of dose curves at different heights in (**A**) 50 and (**B**) 60 s

A total of 40 thermoluminescent personal dosimeters were arranged in the field. According to the site conditions, we conducted certain analyses on the values measured, and obtained the dose distributions at the accident site, as shown in Figs 4 and 5.

As seen from Fig. 4, the dose from the middle to the two sides gradually decreases, which corresponds to the expected results. The closer to the center of the beam, the greater the radiation dose is. At symmetrical positions, the dose on the left is typically greater than the dose on the right, possibly due to an auxiliary device comprised of iron on the left side of the accident site, which increases the scattering of electron rays and toughness radiation.

In Fig. 5, for lower heights at the same position, the dose value increases. Increasingly close to the origin, the dose difference increases, which may be due to the downward exposure of the main beam and the strong scattering of the iron material on the ground. The dose (in 60 s) of 8 cm at −0.5 m should be higher than those at other heights (30 cm 85 cm and 165 cm), but the value measured is significantly lower than those of other heights. We determined that negative sensitivity of the dosimeter occurred due to the high radiation dose. The value of the dosimeter may become lower when it exceeds saturation.

According to the radiation distribution on site , we determined that the main dose of 10 different irradiation points at four heights were relatively concentrated in the middle of the location of the beam within approximately −2 meters and + 2 meters. According to the irradiation dose distribution curves, we calculated the doses for the organs and tissues and the effective doses estimated for the tissues and organs.

### Dose estimation results

When the electron beam is calibrated, the TLD is covered with 1 mm polyethylene plastic. The half-value layer of 2.2 MeV electron was about 1 mm in ABS plastic and polyethylene plastic. The thicknesses of these materials is equal to five half-value layers. The skin absorbed dose formula is as follows:

(1) \begin{equation*} \mathrm{Dp}(0.07)=\left({D}_{60}-{D}_{50}\right)\times 0.4\times{2}^5 \end{equation*}

where *D*_50_ and *D*_60_ are the doses in 50 s and 60 s, respectively. The estimated dose results are shown in Table 1. The uncertainty includes the class A uncertainty and the uncertainty of the calibration factor.

**Table 1:** Dose estimation results for skin (Gy)

Skin parts of the body	Dose in 50 s (Gy)	Dose in 60 s (Gy)	Dose between 50 and–60s (Gy)	Irradiation dose (Gy)	Uncertainty (Gy)
Thyroid gland	2.08	3.01	0.93	11.9	4.7
Upper arm	4.30	5.30	1.00	12.8	5.1
Left side of the body	4.18	5.07	0.89	11.4	4.5
Thigh	7.97	9.05	1.08	13.8	5.5
Upper calf	9.41	10.68	1.27	16.3	6.5
Lower calf	10.73	11.55	0.82	10.5	4.2
Feet	10.96	11.38	0.42	5.4	2.1
Mean skin dose	−	−	−	11.7	4.7

Because the maximum acceptable dose for TLD is 10 Gy, the dosimeters will saturate after the electron beam dose exceeds this limit. As seen from Table 1, the irradiation doses (Gy) to the lower calf and feet were lower than those to the upper calf and other skin areas. The maximum dose was expected on the feet and calf, since the dose decreased with increasing height position. The skin dose measurement results indicated that the skin dose below the calf was higher than the skin dose above the calf for both 50 s and 60 s. However, the estimated dose of the lower leg and foot was lower than the dose of the upper leg. This is due to the small difference between the 50 s and 60 s caused by the superlinearity of the dose. We know that the doses of the foot and lower leg should be at least 16.3 Gy. Thus, we modified the doses of the calf and foot skin. The revised doses estimation results for skin are presented in Table 2.

**Table 2:** Revised dose estimation results for skin (Gy)

Skin parts of the body	Dose in 50 s (Gy)	Dose in 60 s (Gy)	Dose between 50 and 60 s (Gy)	Irradiation dose (Gy)	Uncertainty(Gy)
Thyroid gland	2.08	3.01	0.93	11.9	4.7
Upper arm	4.30	5.30	1.00	12.8	5.1
Left side	4.18	5.07	0.89	11.4	4.5
Thigh	7.97	9.05	1.08	13.8	5.5
Upper calf	9.41	10.68	1.27	16.3	6.5
Lower calf	10.73	11.55	0.82	16.3	6.5
Foot	10.96	11.38	0.42	16.3	6.5
Mean skin dose	−	−	−	14.1	5.6

The lens of the human eye is sensitive to exposure to ionizing radiation. We used the formula below to calculate the dose to the lenses:

(2) \begin{equation*} \mathrm{Dp}(3)=\left(\left({D}_{60}-{D}_{50}\right)\times 0.4\right)/2 \end{equation*}

The results of dose estimation for the lenses are given in Table 3. The mean dose to the eyes was 0.18 ± 0.07Gy. The dose to the left eye was higher than that to the right eye, which was consistent with the actual conditions.

**Table 3:** Dose estimation results for lenses

Location	Dose in 50 s (Gy)	Dose in 60 s (Gy)	Dose difference between 60 s and 50s (Gy)	Estimated dose (Gy)	Uncertainty (Gy)
Lens of the left eye	5.45	6.40	0.95	0.19	0.08
Lens of the right eye	3.71	4.50	0.79	0.16	0.06
Mean lens dose	−	−	−	0.18	0.07

According to the ICRP 2007 recommendations [8], the systemic effective dose is the weighted sum of 14 tissues or organs and the remaining tissues (a total of 15 tissues). We used the following formula to arrive at the total effective dose estimated for the organs [9].

(3) \begin{equation*} E=\sum_T{\omega}_T\times{H}_T=\sum_T{\omega}_T\sum_R{\omega}_R{D}_{T,R} \end{equation*}

where *E* is the effective dose, ω_T_ is the tissue weighting factor for tissue T and ∑ω_T_ = 1. When the type of radiation is electrons, ω_R_ = 1. The results for the organs and tissues were estimated as follows:

(4) \begin{equation*} {D}_{\mathrm{T}}=\left({D}_{60}-{D}_{50}\right)\times 0.4 \end{equation*}

The maximum organ dose was 0.041 Gy, and the maximum tissue weight factor was 0.12. Finally, the total effective dose estimated for the organs was 0.21 Sv. The organs and tissues estimated results are as shown in Table 4.

**Table 4:** Effective dose estimation results for organs and tissues (Gy)

Location	Dose in 50 s (Gy)	Dose in 60 s (Gy)	Dose difference between 60 s and 50 s (Gy)	Effective cumulative doses of 50 s–60 s (Gy)	Effective dose (mSv)
Left side of the gonad	0.237	0.339	0.102	0.041	3.26
Right side of the gonad	0.066	0.094	0.028	0.011	0.90
Left lower quadrant	0.196	0.299	0.103	0.041	4.94
Middle of the lower abdomen	0.113	0.164	0.051	0.020	2.45
Left upper quadrant	0.192	0.287	0.095	0.038	4.56
Right upper quadrant of the abdomen	0.073	0.105	0.032	0.013	1.54
Liver	0.181	0.282	0.101	0.040	1.01
Left lung	0.161	0.232	0.071	0.028	3.41
Heart	0.094	0.143	0.049	0.020	2.35
Thyroid gland	0.060	0.088	0.029	0.012	0.46

## DISCUSSION

Following the radiological accident on 7 July 2014 at Tianjin, a physical dosimetric reconstruction was performed using TLDs. According to this simulated test, the estimated skin dose was 11.9–16.3 Gy, and the average estimated skin dose of the workers was approximately 14.1 ± 5.6 Gy. However, there were errors in the reconstruction of the skin dose, in particular in the process of correcting the calf and foot skin doses. The estimated mean dose for their eye lenses was 0.18 Gy and the estimated effective dose for their organs was 0.46–4.94 mSv; the estimated total effective dose was 0.21 Sv. It can be seen from the dose estimation results that the absorbed dose of the skin was higher than that of other tissues or organs.

Since a TLD is a relative measurement method, the accuracy of dose measurement by a TLD is largely dependent on the accuracy of the calibration method, with certain errors. Moreover, in the process of dose reconstruction estimation, the position of the phantom is fixed, so there are certain errors in the dose estimation.

There are many physical methods for dose reconstruction and estimation, such as experimental physical reconstruction, electron paramagnetic resonance (EPR) measurements, Monte Carlo simulations [11], numerical reconstructions [3, 10, 12] and direct calculation of dose estimation. The best method can be chosen according to the actual situation.

A quick and accurate physical dosimetric calculation of the patient is crucial for clinical treatment. Through physical dosimetric reconstruction results, it can be shown that the main damage to the victim was skin damage. In cases of electron beam exposure, tissue damage may occur even if the effective dose to the whole-body is lower than the dose limit. A special limit on local skin dose, for example, addresses this type of situation for exposure to weakly penetrating radiation [9].

In this accident, operators lacked the necessary safety awareness and knowledge of radiological protection and did not understand or comply with operating procedures. Therefore, radioactive work units should conduct the safety and radiation protection training necessary for personnel engaged in radiation work. Only qualified personnel should be on duty, and a sound radiation protection management system should be established.
